# Opposing role of phagocytic receptors MERTK and AXL in Progranulin deficient FTD

**DOI:** 10.1038/s42003-025-08368-2

**Published:** 2025-07-01

**Authors:** Claire Dudley Clelland, Li Fan, Rowan Saloner, Jon Iker Etchegaray, Chad Richard Altobelli, Sally Salomonsson, Alisha M. Maltos, Aradhana Sachdev, Jingjie Zhu, Se-In Lee, Yaqiao Li, Yungui Zhou, David Le, Chao Wang, Gillian Carling, Lay Kodama, Faten Sayed, Jaun A. Perez-Bermejo, Ethan G. Geier, Jennifer S. Yokoyama, Howie Rosen, Alissa L. Nana, Salvatore Spina, Lea T. Grinberg, William W. Seeley, Fanny Elahi, Adam L. Boxer, Michelle R. Arkin, Li Gan

**Affiliations:** 1 Weill Institute for Neurosciences, University of California San Francisco, San Francisco, CA USA; 2 Memory & Aging Center, Department of Neurology, University of California San Francisco, San Francisco, California USA; 3 Helen and Robert Appel Alzheimer’s Disease Research Institute, Brain and Mind Research Institute, Weill Cornell Medicine, New York, NY USA; 4 Gladstone Institutes, San Francisco, CA USA; 5 Department of Pharmaceutical Chemistry and Small Molecule Discovery Center; University of California, San Francisco, CA USA

**Keywords:** Microglia, Gene expression, Dementia, Genetics of the nervous system

## Abstract

Genetic mutations in the progranulin gene, *GRN*, cause frontotemporal dementia and a lysosomal storage disorder. Using single-nuclei RNA sequencing of the post-mortem brain tissue from adult heterozygous pathogenic granulin variant (*GRN*+/−) carriers we find dysregulation of microglia, phagocytosis and the phagocytic receptors MERTK and AXL. Exogenous progranulin regulates *MERTK* and *AXL* RNA expression in human microglia induced from iPSCs irrespective of *GRN* mutation status, without directly binding to MERTK or AXL proteins. We generated double knock-out mice and find that constitutive homozygous loss of *Grn* and *Mertk* (*Grn*−/−;*Mertk*−/−) rescued microglial disease signature while constitutive homozygous loss of *Grn* and *Axl* (*Grn*−/−;*Axl*−/−) worsened the microglial disease signature and increased lipofuscin. Lower CSF MERTK but not AXL is associated with lower progranulin levels. Furthermore, CSF MERTK is lower in symptomatic but not presymptomatic FTD patients with genetic mutations (*GRN, C9ORF72, and MAPT)* whereas AXL does not change between disease state and control. These data explain in part the inflammation seen in *GRN*-FTD and are applicable to other inflammatory states in which PGRN, MERTK and AXL play regulatory roles beyond neurodegenerative diseases. The interaction between *GRN, MERTK,* and *AXL* opens potential new therapeutic avenues to intervene on this inflammatory axis.

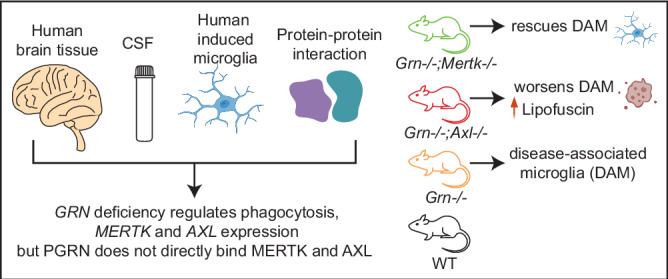

## Abstract (web summary)

Human, mouse and iPSC-derived microglia demonstrate that interaction between GRN and phagocytic receptors can rescue (MERTK) or worsen (AXL) FTD-disease features. CSF MERTK maybe a biomarker of symptomatic disease conversion in genetic FTD.

## Introduction

MERTK and AXL are the phagocytic receptors of macrophages and microglia^1,2^ responsible for maintaining homeostasis in response to apoptosis or inflammatory insults^2–4^. MERTK is constitutively expressed and is especially important for tethering and clearing apoptotic cells under homeostatic states^4^. AXL is dynamically upregulated in response to inflammatory stimuli, and, to a greater degree than MERTK, inhibits Toll-like receptor and cytokine signaling^1,4^. MERTK and AXL have gained interest in neurodegeneration for several reasons: AXL is upregulated in response to disease, including Alzheimer’s pathology^2,4–7^, whereas MERTK is more abundant in homeostatic microglia^2^ and has been shown mediate phagocytosis of alpha-synuclein^8^. In addition, AXL regulates APOE^9^, a key protein and risk factor gene in Alzheimer’s pathogenesis, and both MERTK and AXL are co-expressed with TREM2^10^, another genetic risk factor gene for Alzheimer’s disease (AD). Overall, the emerging evidence suggests that dysregulation of MERTK and AXL is a feature broadly shared by neurodegenerative disease and neuroinflammation. We report here our investigations into another dementia-causing gene, *GRN*, and identify phagocytic receptors MERTK and AXL as part of the pathogenesis of *GRN* dysregulation.

Heterozygous mutations in *GRN*, the gene encoding progranulin (PGRN), are among the leading known genetic causes of frontotemporal dementia (FTD), accounting for approximately one-third of genetic FTD cases and 5-10% of all FTD diagnoses^11–14^. Rare homozygous loss-of-function mutations in *GRN* cause neuronal ceroid lipofuscinosis, a lysosomal storage disease with onset in young adulthood leading to premature death^15^. PGRN deficiency causes microgliosis, increased phagocytosis and neurotoxicity due in part to complement secretion^16–22^. Treatment with recombinant progranulin or increasing endogenous progranulin protects against *GRN-*FTD^23–25^, other dementias^21,26^ and disease states such as stroke^26,27^ in model systems. While PGRN replacement appeals as a viable therapeutic strategy for *GRN*-FTD, too much PGRN may be deleterious. Increased levels of PGRN occur in inflammatory states and tissue injury, including sepsis^28–31^, diabetes^32,33^ and cancer^34–38^. The ideal therapeutic approach would be to tune PGRN and its downstream interactors to target particular disease states. To do so, we require a better understanding of the pathways regulated by PGRN.

Our exploration of single-nucleus RNA sequencing of post-mortem human brain tissue from FTD patients with *GRN* +/− mutations revealed neuroinflammation and dysregulation of phagocytic pathways. Here, we explore the interaction between *GRN*, *MERTK,* and *AXL* in human post-mortem tissue and human CSF from FTD-gene carriers, human iPSC models, biochemical assays and double knock-out mouse models. Results from these studies implicate an interaction between *GRN* deficiency with *MERTK* and *AXL* that underlies the microgliosis and the inflammatory state seen in *GRN*-deficient disease.

## Results

### Post-mortem brain tissue from PGRN-deficient FTD patients shows evidence of dysregulated phagocytosis

**Fig. 1:**
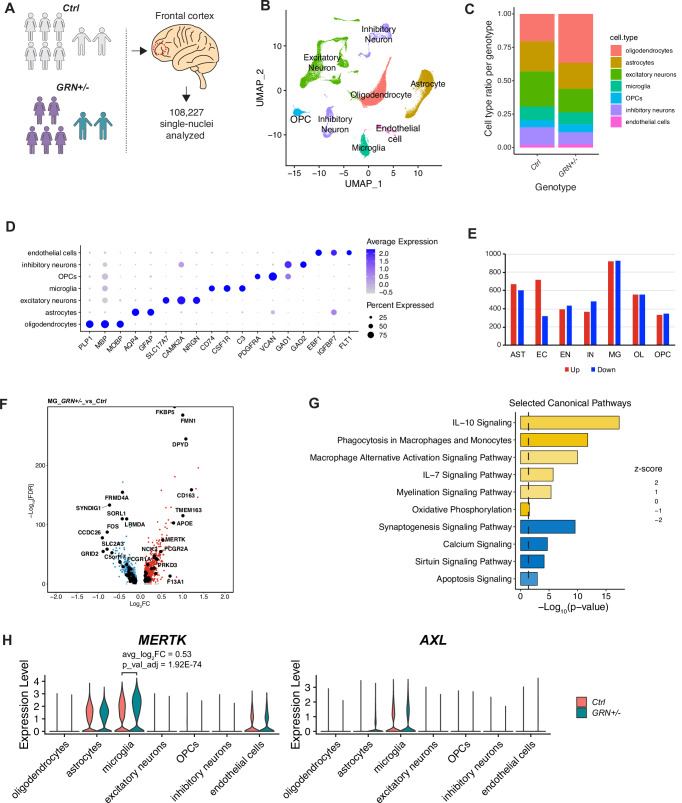
Dysregulated phagocytic pathways in post-mortem *GRN*+/− FTD microglia. **A** Sex and genotypes of human donors used for snRNA-seq. 108,227 nuclei were isolated from the superior frontal cortex of patients with *GRN*+/− FTD (n = 8) and *GRN*+*/+* controls without neurologic disease (Ctrl; n = 7). **B** Uniform Manifold Approximation and Projection (UMAP) plot of all single nuclei and their annotated cell types. OPC = oligodendrocyte precursor cells. **C** Proportion of cell types by genotype. **D** Dot plot showing expression of cell marker genes for each cell type. **E** Number of up-regulated (red) and down-regulated (blue) differentially expressed genes (DEGs) between *GRN-*FTD versus non-FTD control samples for each cell type. FDR < 0.05, log2FC cutoff 0.1. **F** Volcano plot of significant DEGs (FDR < 0.05) between *GRN-*FTD and non-FTD control samples in microglia. **G** Bar plots of Ingenuity Canonical pathways enriched in DEGs identified in (F). P-values were calculated using right-tailed Fisher’s exact test with threshold of significant enrichment as p-value  ≤  0.05 (indicated by a dashed line of −log (p-value)  =  1.3). **H** Violin plot showing expression of MERTK and AXL in all cell types.

We performed single-nuclei RNA sequencing on 108,227 nuclei from superior frontal gyrus from 7 patients with *GRN*+/− haploinsufficiency mutations and 8 *GRN*+*/+* aged controls without neurological disease (Fig. 1A, patient information in Supplementary Data 1). While the proportions of most cell types were similar between the two groups, including excitatory neurons, astrocytes, oligodendrocyte precursor cells (OPCs) and endothelial cells, there was a higher proportion of oligodendrocytes and a lower proportion of microglia and inhibitory neurons in the FTD patients (Fig. 1B–D). All cell types isolated showed large numbers of differentially expressed genes between *GRN-*FTD and control patients (Fig. 1E, Supplementary Data 2). We were particularly intrigued by the difference in microglia given that microgliosis and neuroinflammation are well-established pathologic features of *GRN*-FTD, compared to other types of genetic FTD and to sporadic FTD^39–42^. Microglia in *GRN*-FTD showed a gene-expression profile consistent with disease state microglia^43,44^, including increased expression of *APOE, CTSB, TLR2, RPS19, MYO1E*, etc. (Fig. 1F, Supplementary Data 2). The differences in microglia expression patterns mapped to an upregulation in pathways involved in phagocytosis and inflammatory signaling (Fig. 1G). In addition to dysregulated phagocytic pathways, we noted that IL-10 signaling was the highest upregulated pathway, and that IL-7 signaling was also upregulated. IL-10 and IL-7 are known to regulate MERTK and AXL phagocytic receptors^45–50^. Indeed, we found that *MERTK* was significantly transcriptionally upregulated in *GRN-*FTD (Bonferroni corrected p = 1.9E-74) compared to control brain; however *AXL* expression did not significantly differ between the two conditions (Bonferroni corrected p = 0.19) (Fig. 1H).

### Human post-mortem *GRN-*FTD microglia have a distinct transcriptional profile

**Fig. 2:**
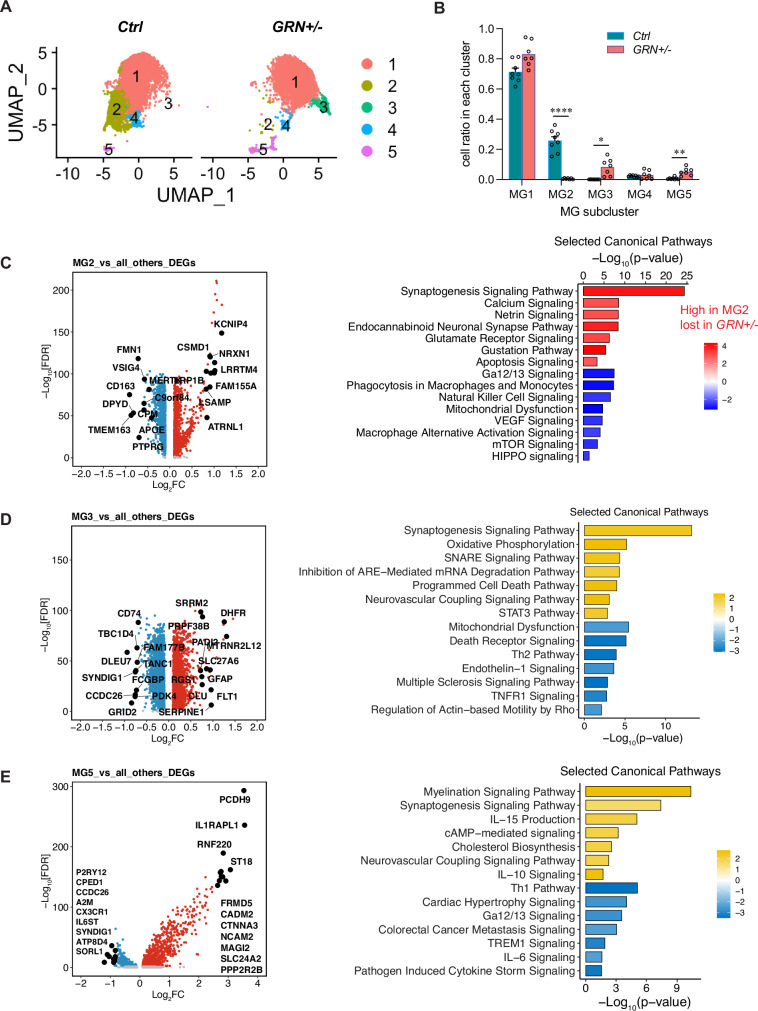
*GRN*+/− FTD microglia have a distinct transcriptional profile. **A**, **B** UMAP plots of microglia from *GRN-*FTD and non-FTD controls show the emergence (MG3, MG5) and loss (MG2) of distinct subclusters. **C** Volcano plot of differentially expressed genes (DEGs, left) and bar plot of top Ingenuity Canonical pathways (right) of MG2 subcluster vs. all other microglia subclusters. Note that because the MG2 subcluster was essentially lost in *GRN-*FTD, both the up- and down-regulated pathways were also lost. **D**, **E** Volcano plots (left) and bar plots (right) of MG3 (D) and MG5 (E) pathways that emerged in *GRN*-FTD microglia. Cut offs for inclusion: |Log2FC| >  0.1 and FDR < 0.05. Two-tailed, unpaired t test, *p < 0.05, **p < 0.01, ***p < 0.001, ****p < 0.0001.

We further investigated the microglia profile in *GRN-*FTD by comparing the transcriptional signatures of microglia subclusters (Fig. 2A, Supplementary Data 3). The largest subcluster in both *GRN-FTD* and control microglia (MG1) represents homeostatic microglia (Fig. 2A, B), which was similar between the two groups. *GRN-*FTD patients displayed the emergence of two subpopulations (MG3, MG5) that were nearly absent in control brains (Fig. 2B). The differences between *GRN-*FTD and controls (absence of MG2 and emergence of MG3, MG5) indicated that the transcriptional upregulation of phagocytic pathways between *GRN-*FTD and controls we observed earlier (Fig. 1) is due to a change in a subset of microglia in the diseased state. Loss of MG2 microglia may release a brake on phagocytosis, as MG2 (present only in controls) downregulate phagocytic pathways (Fig. 2C). This effect may be exacerbated by an upregulation in microglia subpopulations MG3 and MG5 only seen in *GRN-*FTD patients. These subpopulations showed an upregulation of programmed cell death and dysregulation of regulators of the inflammatory response, including TNFR1, STAT3 (MG3, Fig. 2D), and IL-10, IL-15 and IL-6 (MG5, Fig. 2E) which can cause tissue damage when aberrantly expressed. Furthermore, we found significant changes in synapse signaling across all altered microglia subclusters (Fig. 2C-E). Altered phagocytosis and synapse signaling in combination with neuroinflammation aligns with animal studies showing that loss of PGRN leads to increased neuronal synaptic pruning and excitatory neuronal loss through dysregulated inflammation^19,20^.

### PGRN regulates *MERTK* and *AXL* expression in human iPSC-induced microglia but does not bind directly to MERTK or AXL proteins

**Fig. 3:**
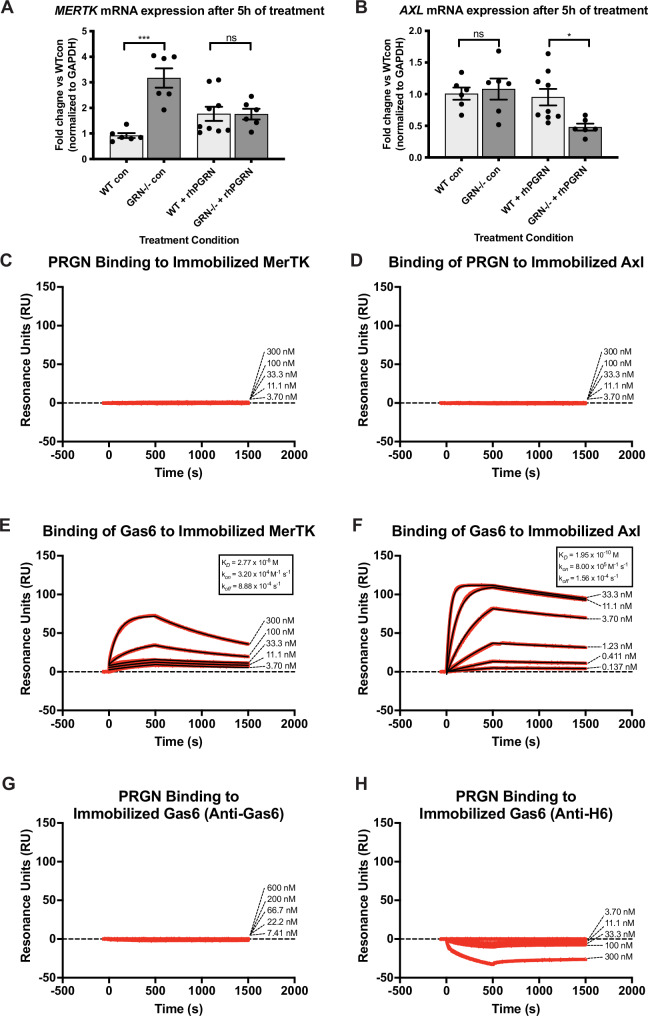
Progranulin regulates *MERTK* and *AXL* RNA expression but binds neither MERTK nor AXL proteins. **A** The upregulation of *MERTK* RNA in *GRN−/−* relative to control human induced microglia (Sidak’s multiple comparison test p < 0.0001) was normalized by exogenous administration of 100 nM progranulin (Sidak’s multiple comparison test p < 0.98). **B** While there is no baseline difference in *AXL* mRNA expression in untreated WT or *GRN*−/− microglia (Sidak’s multiple comparison test p = 0.91), 100 nM exogenous progranulin significantly decreased *AXL* RNA expression in *GRN*−/− but not WT human induced microglia (Sidak’s multiple comparison test p < 0.05). **C**, **D** We found no interaction of PGRN over a wide range of concentrations with either MERTK or AXL by surface plasmon resonance (SPR). **E**, **F** As controls, we show that Gas6, a known binder of MerTK and Axl, binds these receptors with 27.7 nM and 193 pM affinities respectively and theoretical Rmax of 135 RU. Data representative of three experiments (red) were fit to a 1:1 kinetic model (black) to obtain binding parameters. We also find no binding of PGRN to GAS6 after immobilizing GAS6 with an anti-GAS6 antibody (**G**) or H6-tagged-GAS6 antibody (**H**), which anchor Gas6 at different sites. The inverted sensograms in (H) suggests nonspecific binding between PGRN and the Anti-H6 reference surface. Error bars = SEM. *p < 0.05, ***p < 0.001.

Given the dramatic transcriptomic changes seen in microglia from *GRN-*FTD patients that point to dysregulation of phagocytosis, we decided to focus on two microglial phagocytic receptors, MERTK and AXL, and their potential interactions with PGRN. To investigate these interactions, we first measured mRNA levels of *MERTK* and *AXL* in microglia derived from *GRN* knock-out (KO)^25^ or WT iPSCs. *MERTK* was upregulated (Fig. 3A) whereas *AXL* mRNA expression was not altered in *GRN*-KO compared to WT microglia (Fig. 3B), recapitulating the transcriptomic findings from the brain tissues (Fig. 1H). After exogenous progranulin administration, both *MERTK* and *AXL* were downregulated in the *GRN-KO* microglia, but not the WT microglia (Fig. 3A, B). Indeed, exogenous PGRN normalized *MERTK* expression in *GRN*-KO (Fig. 3A). In combination with the upregulation of *MERTK* in *GRN*-FTD (Fig. 1H), these results indicate that PGRN keeps *MERTK* transcriptionally repressed, whether it is supplied endogenously or exogenously. In contrast, loss of PGRN had no effect on *AXL* expression and PGRN only represses *AXL* when provided exogenously to *GRN*-deficient microglia.

We next asked whether PGRN directly binds MERTK or AXL at the protein level. We measured the interaction of purified human proteins by surface plasmon resonance (SPR). We found that PGRN bound neither MERTK (Fig. 3C) nor AXL (Fig. 3D), even at high concentrations. In contrast, GAS6, a known ligand of MERTK and AXL, exhibited expected binding to MERTK (Fig. 3E) and AXL (Fig. 3F) at physiologically relevant concentrations of their known ligand. We then questioned whether PGRN interacts with MERTK or AXL indirectly, by binding their ligand GAS6, as has been previously reported^51^. In contrast to this previous report, we found no interaction between PGRN and GAS6, using four complementary methods to confirm the result: direct antibody immobilization of GAS6, immobilization of via a His-tag on GAS6 (Fig. 3G, H) SPR competition assay (Supplementary Fig 1A,B**)** and immunoprecipitation (Supplementary Fig 1C). These data indicate that the regulation of *MERTK* and *AXL* at the transcriptional level by progranulin is unlikely to result from direct interaction between PGRN and MERTK or AXL proteins.

### Loss of *Mertk* rescues microglial disease signature in *Grn* KO mice, whereas loss of *Axl* worsens microglia disease phenotype

To examine potential functional interactions of PGRN with MERTK and AXL in an in vivo setting, we created double KO mice with homozygous loss of both *Grn* and *Mertk* (Grn−/−; *Mertk*−/−) or homozygous loss of both *Grn* and *Axl* (Grn−/−; *Axl*−/−). Each line was created on the same parental C57Bl/6 background. We compared brains from adult mice of both sexes, aged 10–12 months, from each of the double KO mouse lines to WT control mice and to single KO mice (*Grn*−/−, *Mertk−/−* and *Axl*−/− mice). At baseline *Grn−/−* mice did not show a difference in Mertk or Axl mRNA or protein (Supplementary Fig 2).

**Fig. 4:**
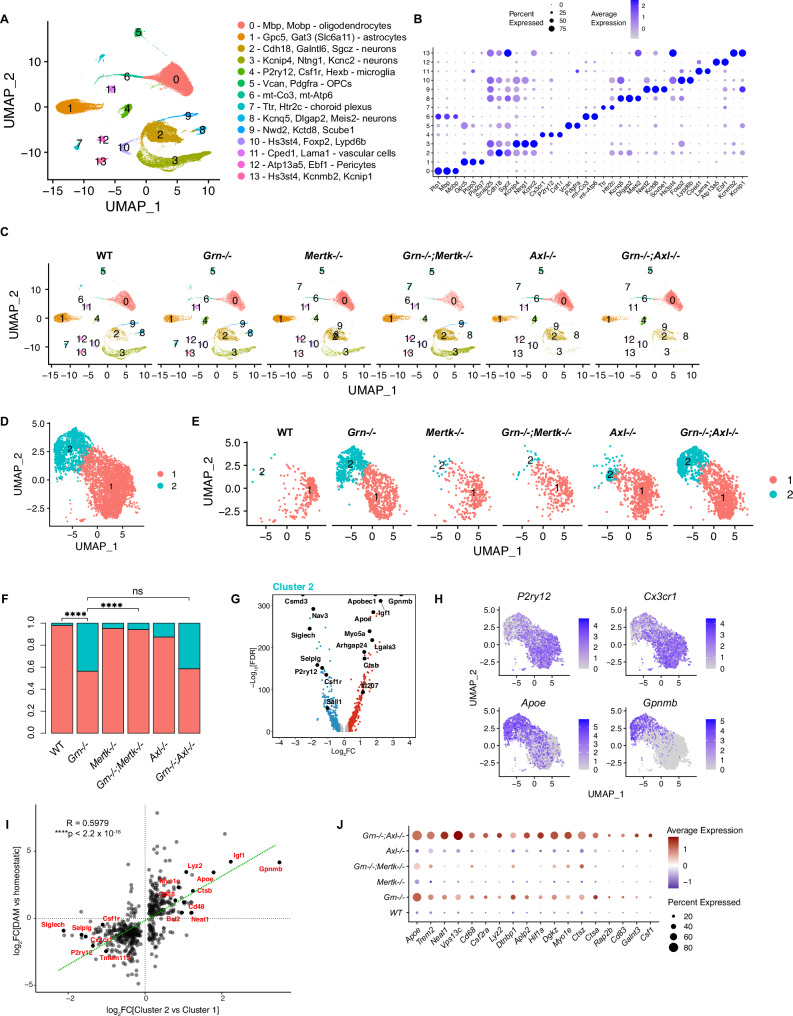
Loss of *Mertk* rescues microglia signature in *Grn* knock-out mice, whereas loss of *Axl* worsens it. We sequenced 166,224 single-nuclei across all mice. **A** UMAP plot of all single nuclei and their annotated cell types with their top marker genes. OPC = oligodendrocyte precursor cells. **B** Dot plot showing expression of cell marker genes for each cell type. **C** UMAP plots by genotype. Note cluster 3 neurons are lost in *Axl−/−* and *Grn*−/−;*Axl*−/−. **D**, **E** UMAP plot of all 4668 microglial cells analyzed and reclustered into homeostatic (cluster 1, salmon) and disease-associated (cluster 2, blue) cells. **E** UMAP of microglia by genotype. **F** Proportion of cells in clusters 1 and 2 by genotype. ****p < 0.0001, two-sided Fisher’s exact test. ns = not significant. **G** Volcano plot of DEGs defining cluster 2 compared to cluster 1. Cutoffs for inclusion: |Log2FC| > 0.1 and FDR < 0.05. **H** Feature plots of transcript expression overlaid onto UMAP of all microglial cells, for example homeostatic markers (*P2ry12, Cx3cr1*) and disease-associated markers (*Apoe, Gpnmb*) markers. Colored scale bar denotes normalized expression level. **I** Scatterplot showing the correlation between our comparison of DEGs in the microglial cluster 2 versus cluster 1 (x axis) and a previously published^44^ comparison of disease-associated microglia (DAM/MGnD) versus homeostatic microglia (y axis). R = 0.5979, ****p < 2.2 × 10^−16^, Pearson’s correlation. **J** Dot plot showing that disease-associated genes upregulated in *Grn−/−* relative to WT microglia are further upregulated in *Grn−/−;Axl−/−*. This effect is driven by interaction between loss of *Grn* and loss of *Axl* as *Axl−/−* does not show this profile. By contrast, *Grn−/−;Mertk−/−* rescues the *Grn*−/− induced profile. Expression average denoted by color, percent of microglia expressing this marker denoted by circle size.

We first performed single-nuclei RNA-sequencing on 166,224 thalamic cells from each of the 6 genotypes (n = 4 per group) (Fig. 4A,B, Supplementary Data 4). We chose the thalamus as prior work^18,19,52^ indicated that the thalamus is a key site of dysfunction in this PGRN KO model. *Mertk* KO did not significantly alter any of the major subclusters (Fig. 4C). Interestingly, *Axl* KO resulted in the near complete loss of an abundant subcluster of *Kcnip4, Ntng1, Kcnc2* interneurons (cluster 3 interneurons, Fig. 4C).

To understand the different effects of *Mertk* and *Axl* on microglia states in the presence or absence of *Pgrn*, we examined microglial subclusters across genotypes (Fig. 4D-F). We found two distinct subclusters of microglia: one corresponding to homeostatic microglia (salmon) and another corresponding to diseased microglia (blue) (Fig. 4D-F, Supplementary Data 5). Homeostatic microglia were the predominant population in WT thalamus (Fig. 4E, F). As expected, a distinct population of diseased microglia emerged in *Grn* KO, and were defined by loss of homeostatic markers (*P2ry12, Siglech, Csf1R, Sall1*) and induction of disease state markers (*Apoe, Gpnmb, Ctsb, Igf1*) (Fig. 4G, H). These microglia are consistent with previous descriptions of disease-associated microglia (DAMs) described in mouse models of Alzheimer’s disease^44^ (Fig. 4I**)**. *Mertk* KO had no effect on the ratio between diseased and homeostatic microglia (Fisher’s exact test, p < 0.0001), while *Axl* KO slightly increased the proportion of diseased microglia compared to WT (Fisher’s exact test, p < 0.05) (Fig. 4E, F). In contrast, ablation of *Mertk* or *Axl* resulted in substantial differences in the PGRN deficient microglia populations. Notably, ablation of *Mertk* in *Grn−/−* reduced the abundance of diseased state (cluster 2) relative to homeostatic state (cluster 1) microglia to WT control levels (Fisher’s exact tests: *Grn*−/−;*Mertk*−/− vs *Grn*−/− p < 0.001, *Grn*−/−;*Mertk*−/− vs *WT* p = 0.30) (Fig. 4E,F), indicating that *Mertk* KO rescues the *Grn* KO phenotype. This rescue was confirmed by a reduction in expression of disease markers (Fig. 4J). By contrast, although the ratio of disease-associated to homeostatic microglia was similar between *Grn−/−* and *Grn*−/−;*Axl*−/− (Fisher’s exact test, p > 0.99) (Fig. 4E, F), the disease-associated RNA expression was further upregulated in *Grn*−/−;*Axl*−/− (Fig. 4J). Thus, *MertK* KO reduces while *Axl* KO enhances the disease states in *Grn* KO microglia.

**Fig. 5:**
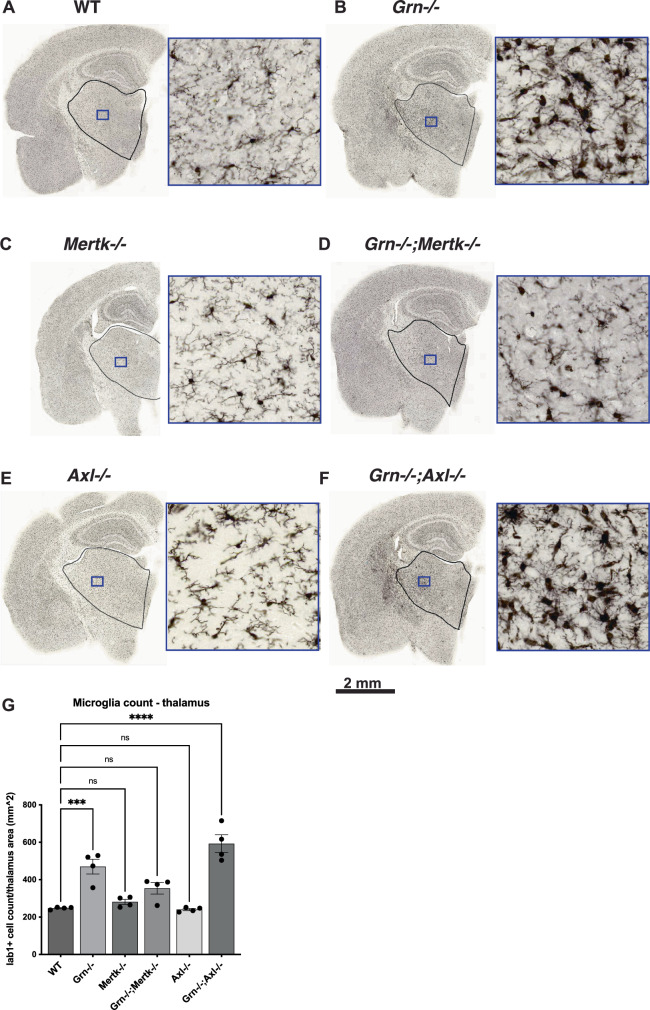
Ablation of *Mertk*, but not *Axl*, rescues thalamic microgliosis in *Grn−/−* mouse. **A**- **F** Representative hemibrain images strained with anti-Iba1 microglia marker from the 6 genotypes with thalamic regions outlined. Inset (blue box) is depicted to illustrate differences in microglia morphology. **G** *Grn−/−* increased Iba1+ microglia count compared to WT (Tukey’s multiple comparison test p < 0.001) whereas *Grn−/−;Mertk−/−* restored microglia counts to WT baseline (Tukey’s multiple comparison test, p = 0.14). *Grn−/−;Axl−/−* exacerbated microgliosis compared *Grn−/−* (one-way ANOVA F(5,18) = 24, p < 0.001, Tukey’s multiple comparison test, *Grn−/−; Axl−/−* vs WT p < 0.0001*, Grn−/−; Axl−/−* vs *Grn−/−* p < 0.05). ns not significant. Error bars = SEM.

We confirmed the effect of *Grn*, *Mertk* and *Axl* gene KO on thalamic microglia count using immunocytochemistry (Fig. 5). In concordance with our single-cell transcriptomic profiling (Fig. 4) and prior histological analysis of the progranulin KO mouse^19,52^, Iba1+ microglia count was increased in *Grn* KO mice compared to WT controls (one-way ANOVA F(5,18) = 24.0, p < 0.001; Tukey’s post-hoc test WT vs *Grn*−/− p < 0.001) (Fig. 5A, B–G). Knock-out of *Mertk* or *Axl* alone did not alter microglia count compared to WT controls (Tukey’s post-hoc test WT vs *Mertk*−/− p = 0.95, WT vs *Axl*−/− p > 0.99) (Fig. 5C,E–G). Ablation of *Mertk* in *Grn−/−* microglia restored the microglia count back to WT levels (Tukey’s post-hoc test WT vs *Grn−/−;Mertk−/−* p = 0.14), while ablation of *Axl* failed to rescue microgliosis caused by *Grn*-KO (Tukey’s post-hoc test WT vs *Grn−/−;Axl−/−* p < 0.001) (Fig. 5D, F, G). Furthermore, ablation of *Axl* increased lipofusin accumulation in *Grn−/−;Axl−/−*, but *Axl* ablation alone did not cause lipofuscin accumulation (Supplementary Fig 3).

### Cerebrospinal fluid (CSF) levels of MERTK, but not AXL, are decreased in symptomatic *GRN*-FTD and other genetic forms of FTD

**Fig. 6:**
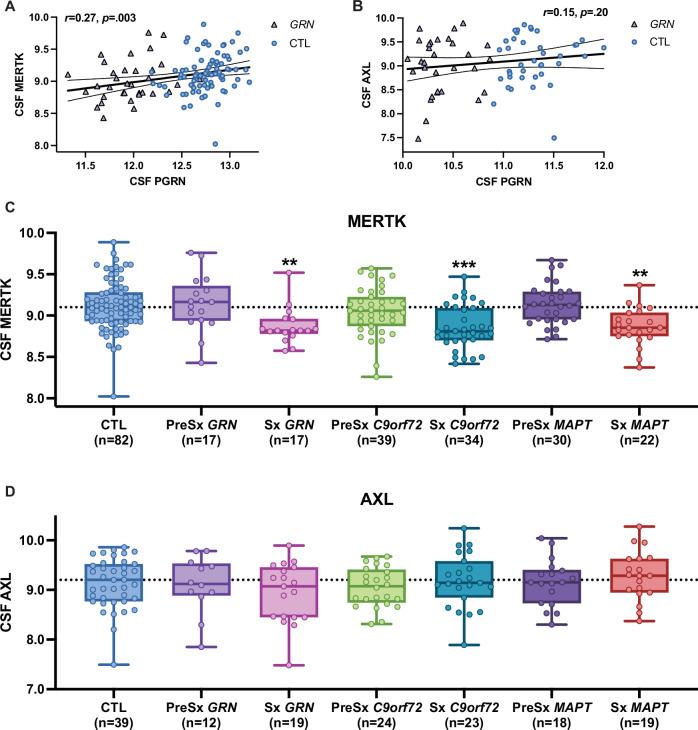
CSF MERTK but not AXL is associated with decreased CSF PGRN and is decreased in symptomatic *GRN*-FTD patients. **A**, **B** Pearson correlations of CSF PGRN with CSF MERTK (A; n = 116) and CSF AXL (B; n = 70) shows that CSF MERTK but not AXL is associated with lower CSF PGRN. Data points are symbol and color-coded by *GRN* mutation carriers (purple triangle) and controls (CTL; blue circle). **C** CSF MERTK is significantly lower in symptomatic (Sx) but not presymptomatic (PreSx) *GRN*, *C9orf72* and *MAPT* mutation carriers compared to controls (CTL) (Kruskal-Wallis p < 0.0001; Dunn’s multiple comparisons test vs CTL **p < 0.01, ***p < 0.001). **D** CSF AXL is not significantly different between groups (Kruskal-Wallis p = 0.66). Horizontal dotted line represents median CSF MERTK or AXL levels in CTL. Units for all aptamers are expressed as log2 RFU (relative fluorescence unit). Box plots represent the median and 25th and 75th percentiles, and box hinges represent the interquartile range of the two middle quartiles within a group. Min and max data points define the extent of whiskers (error bars).

We next analyzed CSF proteomic data from GRN haploinsufficiency mutation carriers and noncarrier controls from the ALLFTD consortium to determine whether levels of MERTK and AXL tracked with presymptomatic and symptomatic stages of *GRN*-FTD. CSF GRN protein, previously identified as the most differentially decreased protein in *GRN* haploinsufficiency mutation carriers compared to controls, exhibited a significant, positive correlation with CSF levels of MERTK (*r* = 0.27, *p* = .003) (Fig. 6A) but not AXL (*r* = 0.15, *p* = 0.20) (Fig. 6B). In group comparisons, we found a significant decrease in CSF MERTK protein levels (one-way ANOVA F(2,113) = 5.4, p = 0.006) in symptomatic *GRN* compared to controls (Tukey’s post-hoc test p = 0.006, Log2FC = -0.25) and presymptomatic *GRN* (Tukey’s post-hoc test p = 0.021, Log2FC = -0.28) (Fig. 6C). In contrast, CSF AXL did not differ across symptomatic *GRN*, presymptomatic *GRN*, and controls (one-way ANOVA F(2,67) = 0.12, p = 0.88) (Fig. 6D).

Given the strong link between CSF MERTK and symptomatic *GRN*-FTD, we next asked whether CSF MERTK exhibited a similar pattern at the symptomatic stage of other genetic FTDs, specifically *C9orf72* and *MAPT* mutation carriers. We found a similar-sized decrease in CSF MERTK in symptomatic *C9orf72* (Tukey’s post-hoc test p < 0.001, Log2FC = −0.26) and *MAPT* (Tukey’s post-hoc test p < 0.001, Log2FC = −0.25) compared to controls (Fig. 6C). AXL did not differ between gene-carriers and controls (Fig. 6D).

Unlike the decrease in MERTK observed in the CSF of symptomatic GRN-FTD patients (Fig. 6C) and human-induced microglia (Supplementary Fig. 4A, B), MERTK levels in post-mortem brain tissue did not differ between GRN-FTD patients and non-carrier controls (Supplementary Fig. 4D, E). In contrast, while AXL levels in mutation carrier CSF (Fig. 6D) and induced human microglia (Supplementary Fig. 4A, C) were comparable to controls, AXL was elevated in post-mortem tissue (Supplementary Fig.s 4F, G). Whether these differences between CSF and post-mortem MERTK and AXL levels reflect distinct subcellular localization during disease progression remains to be investigated.

## Discussion

Evidence of microgliosis in the brain of human *GRN* mutation carriers and animal models of *GRN*-FTD^18–20,52^, to a greater degree than in sporadic and other mutation-driven FTD cases^39–42^, points to microglia as a key pathologic factor in *GRN*-FTD. We recapitulated these findings using single-nuclei transcriptomics from *GRN*+/− mutation carriers (Figs. 1, 2) and in our *Grn* KO animal model (Figs. 4, 5). Drilling down further, we found that key phagocytic receptors and regulators of microglial homeostasis, MERTK and AXL, interact with PGRN in specific and complex ways. Our findings have interesting implications for the development of therapies and biomarkers for *GRN*-FTD.

*PGRN regulates MERTK and AXL but does not bind either directly*. Our studies reported here using human post-mortem tissue and pre-morbid functional measures from *GRN*+/− FTD patients, along with cellular and mouse models of PGRN deficiency identify important interactions between PGRN, phagocytosis and the phagocytic receptors MERTK and AXL. The interaction is not as simple as protein-protein binding, as we find that PGRN binds neither MERTK nor AXL in biochemical assays (Fig. 3, Supplementary Fig 1). In addition, PGRN does not bind GAS6 (Fig. 3, Supplementary Fig 1), which opposes a previous report of direct PGRN-GAS6 binding^51^. During our optimization experiments, we noticed that GAS6 tends to absorb nonspecifically into the SPR flow system and onto sensor surfaces, which creates false positive signal. We added 0.1% BSA as a carrier protein to eliminate nonspecific binding. Furthermore, Fujita et al.^51^ did not report the amount of Gas6 immobilized, which makes it difficult to interpret whether there is physiologic relevance with the PGRN binding they observe. Under our conditions, we did not observe an interaction between PGRN and GAS6. Because of the discrepancy between our findings and previously published findings^51^, we also confirmed the absence of PGRN and GAS6 binding with an SPR competition assay (Supplementary Fig 1A, B) and immunoprecipitation (Supplementary Fig 1C). We are confident that PGRN does not directly bind GAS6, AXL or MERTK under physiologic conditions in these direct protein interaction assays.

Instead, we find that both in post-mortem human samples (Fig. 1H) and in human cell models (Fig. 3A, B), PGRN deficiency leads to transcriptional upregulation of *MERTK* but not of *AXL*. The upregulation in phagocytosis and inflammatory signaling (Fig. 1G) we observe may drive *MERTK* upregulation or be a consequence of *MERTK* upregulation, as these pathways are known to regulate MERTK^1,2,4^. At the protein level, MERTK but not AXL is decreased in CSF from symptomatic *GRN*-FTD patients (Fig. 6C) and is also decreased in human induced microglia (Supplementary Fig 4A, B) but is not different between post-mortem brain tissue from *GRN*+/− and *GRN*+*/+* individuals (Supplementary Fig 4D, E). The difference between CSF and post-mortem MERTK levels could reflect downregulation of MERTK expression, lower secretion or release from the cell, or active retention of MERTK in disease. Whether this lowering of MERTK is compensatory, as suggested by our mouse data, or pathogenic remains to be investigated. Conversely AXL levels from mutation carrier CSF (Fig. 6D) and in induced human microglia (Supplementary Fig 4A, C) are equivalent to controls but AXL is increased in post-mortem tissue (Supplementary Fig 4F, G). We cannot exclude the possibility that post-mortem interval and the death process itself may also influence MERTK and AXL levels.

Because MERTK levels decreased in CSF after symptom onset, but not before, in gene carriers, lower levels of MERTK could indicate the conversion to disease. The differential regulation of MERTK by PGRN and the potential for MERTK as a biomarker would be best studied longitudinally in living humans, which would also tease apart whether changes in MERTK expression are compensatory or pathogenic. Such information is critical to therapies for *GRN*-deficient FTD, but also disease states in which *GRN* is upregulated or overexpressed, such as tissue injury, sepsis, diabetes and cancer. Interestingly, lower levels of CSF MERTK but not AXL differentiated not only symptomatic from presymptomatic *GRN* +/− carriers, but also symptomatic from pre-symptomatic for *MAPT* and *C9ORF72* mutation carriers as well, suggesting that it may be a biomarker of conversion to symptomatic FTD. We hope that our data inspires the testing of CSF-MERTK as a biomarker for onset of symptomatic FTD and the testing of MERTK modulators in *GRN*-FTD.

*Complex interactions between PGRN, MERTK and AXL*. Our mouse data suggests that the interaction between PGRN and MERTK or AXL is inverse; homozygous loss *Mertk* protects against the microglial disease phenotype induced by *Grn* loss alone, whereas homozygous loss *Axl* worsens *Grn−/−* disease phenotype (Figs. 4, 5). It is puzzling that loss of a homeostatic marker (*Mertk*) corrects disease phenotype, and loss of a disease-responsive marker (*Axl*) worsens it; however, the loss of *Mertk* has been shown to be beneficial in other settings. In animal studies of viral infection, homozygous *Mertk* knock-out increased mouse survival. This was attributed to lowering innate anergy through decreased *Mertk-*induced IL-10 and TGF-beta production, thus permitting continued immune attack on the virus^47^. Genetic ablation of astrocytic *Mertk* in a mouse model of social deprivation prevented phagocytosis of excitatory synapses and behavioral symptoms^53^. In our animal models reported here, *Mertk* interaction with *Grn* appears to be a key checkpoint for the formation of disease-associated microglia. Whether MERTK inhibition has therapeutic potential in PGRN-deficient states such as *GRN-*FTD depends on whether disease-associated microgliosis is beneficial or harmful.

Our findings have implications for other disease states, most notably Alzheimer’s disease, but also diabetes, cancer, infection and inflammation in which altered PGRN is known to play a causative or associated role. We showed previously that loss of PGRN accelerates pathologic states in Alzheimer’s disease models^21^, and *GRN* mutations are a risk factor gene for Alzheimer’s disease^54^. In an overexpression AD model (*App/Ps1*), loss of *Mertk, Axl* or both decreased a microglia-mediated pathologic hallmark (dense core plaques)^5,7^. Here we show that disease-associated genes including *APOE*, described in an AD mouse model^44^, are upregulated in both human *GRN-*FTD microglia (Fig. 1) and *Grn*-KO mouse microglia (Fig. 4). This overlap between disease-mechanisms supports common pathways in dementia. We hypothesize that PGRN interactions with MERTK and AXL will have broader implications, including to other disease states yet to be investigated.

*Surprising impact of Axl knock-out on white matter microgliosis and interneurons in mice*. We noted predominant microgliosis in peri-thalamic white matter tracts (fimbria, stria terminalis, cerebral peduncle) (Fig. 5). With the additional finding of myelination pathway dysregulation in our human microglia subcluster analysis (Fig. 2, MG5), these data suggest that Axl regulates oligodendrocyte population and myelination. A detailed study of the interaction with *GRN* and *AXL* deficiency in oligodendrocytes is warranted. Lastly, possibly the most surprising finding is the near complete loss of an abundant population of thalamic interneurons (defined by expression of *Kcnip4, Ntng1, Kcnc2*) driven by homozygous *Axl* knock-out (Fig. 4B, C). This unexpected finding suggests *Axl* is critical to the survival or identity of these cells, an interaction which has not been reported to our knowledge. We and others have previously found that *Grn-*KO mice exhibit hyperexcitability in the striatum^18^ and thalamus, likely due to preferential elimination of inhibitory synapses^19^. The observation that *GRN*-FTD brains specifically lose interneurons aligns with these findings, further supporting the role of PGRN in maintaining inhibitory circuitry. Notably, *AXL* is also expressed in astrocytes, which have been implicated in phagocytosis^55^. This raises the possibility that astrocytic *AXL* contributes to regulation of inhibitory neurons either during development or in the adult brain.

In summary, we show that PGRN regulates *MERTK* and *AXL* RNA levels in human induced microglia without binding to these receptors, that *MERTK* is dysregulated in human brain tissue and CSF from *GRN-*FTD patients, and that concurrent KO of *Mertk* and *Grn* in mice rescues *Grn-*deficient disease-associated microglia phenotype, while concurrent KO of *Axl* and *Grn* worsen it. These findings have applicability not only to *GRN-*FTD but other disease states in which PGRN levels are altered such as AD, stroke, inflammation, sepsis, cancer and more. In addition, the interaction between PGRN with MERTK and AXL opens new therapeutic avenues that warrant further investigation.

## Methods

### Human tissue

Twelve patients with heterozygous *GRN* mutations (age range 56–84 years; n = 3 females, n = 3 males) and 12 control subjects without neurologic disease (age range 60–98; n = 9 females, n = 3 males) were enrolled in the study through the University of California (UCSF) Memory & Aging Center as part of a larger study to catalog the natural history of FTD. All FTD-*GRN* patients included in this study had a pathology confirmed diagnosis of FTLD-TDP-A and a clinical diagnosis of behavioral variant FTD (n = 7), primary progressive aphasia (n = 3) or cortical basal syndrome (n = 1) (Supplementary Data 1). Controls had no clinical neurologic diagnosis. Details regarding incidental pathologic findings according to NIA-AA AD Criteria^56^ are show Supplementary Data 1. The study protocol was approved by the UCSF Committee on Human Research Institutional Review Board. All ethical regulations relevant to human research participants were followed. Written informed consent was obtained from all participants with capacity. For those unable to provide informed consent due to diminished capacity, assent was obtained from the participant and written consent was obtained from a designated surrogate decision-maker. Subjects underwent a standardized neurological evaluation. Participants underwent autopsy and brain donation to the UCSF Neurodegenerative Disease Brain Bank. Blocks, ~0.5–1 cm^2^, of superior frontal gyrus were cut from unfixed, frozen tissue for single cell analysis and protein quantification.

### Human and mouse single-nuclei RNA sequencing (snRNA-seq)

For the human single-cell experiment: 8 *GRN*-FTD and 7 non-FTD controls, 2 males and 5–6 females per group, were included in single-cell analysis based on quality of RNA (RIN > 5). For the mouse single cell experiment: 4 mice from each group (2 males and 2 females) at 10–12 months of age. Nuclei isolation from frozen human superior frontal gyrus and mouse hippocampi was adapted from a previous study^57^ with modifications. All procedures were performed on ice or at 4 °C. In brief, brain tissue was placed in 1 500 µl of Sigma nuclei PURE lysis buffer (Sigma, NUC201-1KT) and homogenized with a Dounce tissue grinder (Sigma, D8938-1SET) with 15 strokes with pestle A and 15 strokes with pestle B. The homogenized tissue was filtered through a 35-µm cell strainer, centrifuged at 600 g for 5 min at 4 °C and washed three times with 1 ml of PBS containing 1% bovine serum albumin (BSA, Thermo Fisher Scientific, 37525), 20 mM DTT (Thermo Fisher Scientific, 426380500) and 0.2 U/µl recombinant RNase inhibitor (Ambion, AM2684). Nuclei were then centrifuged at 600 g for 5 min at 4 °C and resuspended in 350 µl of PBS containing 0.04% BSA and 1× DAPI, followed by fluorescence-activated cell sorting to remove cell debris. The sorted suspension of DAPI-stained nuclei was counted and diluted to a concentration of 1,000 nuclei per µl in PBS containing 0.04% BSA. For droplet-based snRNA-seq, libraries were prepared with Chromium Single Cell 3′ Reagent Kits v3 (10x Genomics, PN-1000075) according to the manufacturer’s protocol. The snRNA-seq libraries were sequenced on a NovaSeq 6000 sequencer (Illumina) with 100 cycles.

### Single nucleus RNA-seq data processing

Gene counts were obtained by aligning reads to the hg38 and mm10 genome with Cell Ranger software (v.3.1.0; 10x Genomics) for human and mouse samples, respectively. To account for unspliced nuclear transcripts, reads mapping to pre-mRNA were counted. Cell Ranger 3.1.0 default parameters were used to call cell barcodes. For human snRNAseq, we further removed genes expressed in no more than 3 cells, cells with unique gene counts over 9000 or less than 300, cells with UMI counts over 50,000 and cells with a high fraction of mitochondrial reads (>5%). For mouse snRNAseq, we further removed genes expressed in no more than 3 cells, cells with unique gene counts over 4 000 or less than 300, cells with UMI counts over 20,000 and cells with a high fraction of mitochondrial reads (>5%). Potential doublet cells were predicted using DoubletFinder for each sample separately, and high-confidence doublets removed. Normalization and clustering were done with the Seurat package v3.2.2 (Stuart, Butler 2019). In brief, counts for all nuclei were scaled by the total library size multiplied by a scale factor (10,000) and transformed to log space. A set of 2 000 highly variable genes was identified with FindVariableFeatures function with vst method. This returned a corrected unique molecular identifier count matrix, a log-transformed data matrix and Pearson residuals from the regularized negative binomial regression model. Principal-component analysis was done on all genes, and t-distributed stochastic neighbor embedding was run on the top 15 principal components. Cell clusters were identified with the Seurat functions FindNeighbors (using the top 15 principal components) and FindClusters (resolution = 0.1). In this analysis, the neighborhood size parameter pK was estimated using the mean variance-normalized bimodality coefficient (BCmvn) approach, with 15 principal components used and pN set as 0.25 by default. Sample integration was performed using FindIntegrationAnchors and IntegrateData functions in Seurat. For each cluster, we assigned a cell-type label using statistical enrichment for sets of marker genes and manual evaluation of gene expression for small sets of known marker genes. Differential gene expression analysis was done using the FindMarkers function and MAST (Finak 2015). To identify gene ontology and pathways enriched in the DEGs, DEGs were analyzed using the MSigDB gene annotation database (Subramanian 2005, Liberzon 2011). To control for multiple testing, we used the Benjamini–Hochberg approach to constrain the FDR.

### Human CSF

CSF levels of AXL and MERTK were measured at baseline in *GRN* mutation carriers and noncarrier controls from patients enrolled in the ALLFTD Consortium, a multi-site longitudinal observational study of genetic FTD^58–60^. Patient cohort characteristics are published^58^. CSF AXL and MERTK data were collected as part of the aptamer-based SomaScan proteomics platform^58,61^. Proteomic data were sourced from two separate versions of the platform, a proprietary version that captured >4k proteins, including AXL, and a commercially-available version that captured >7k proteins, including MERTK. CSF GRN protein was measured across both platforms. The proteomic data processing pipeline is described in detail^58^ elsewhere. Briefly, individual protein levels, expressed as relative fluorescence units, were Log_2_-transformed and regressed for age and sex based on control sample data to account for ‘typical’ demographic effects and isolate disease-specific signals in mutation carriers. CSF AXL data from the 4k platform were available in 31 *GRN* carriers and 39 controls. CSF MERTK data from the 7k platform were available in 34 *GRN* carriers and 82 controls. CSF MERTK data were also examined in other genetic FTD mutation carriers from ALLFTD, specifically 73 *C9orf72* and 52 *MAPT* mutation carriers. Mutation carriers were classified as presymptomatic or symptomatic based on CDR Global scores of 0 (presymptomatic) or ≥0.5 (symptomatic)^58,60^.

### Human induced microglia

PGRN KO iPSCs^25^ were generated and validated by the Conklin lab at Gladstone Institutes in a non-diseased cell line (WTC). We maintained iPSCs in E8 media (Gibco A1517001) plated on Matrigel (Corning 356231), passaging at 60–80% confluency. All cell lines had a normal karyotype and negative quarterly mycloplasma testing. iPSCs were differentiated into microglia following a published protocol^62^ with the following modifications: we used E8 as the induction media and StemPro-34 SFM (Gibco 10639011) as the differentiation/maintenance media. Around day 30, floating microglia precursor cells were collected from the parental flask and further differentiated for two additional weeks as monocultures on uncoated tissue culture plates in RPMI-1640 (Life Tech/Fisher 12633-012) supplemented with 2 mM GlutaMAX-I (Gibco 35050061), 10 ng/ml GM-CSF (PeproTech 300-03), 100 ng/ml IL-34 (PeproTech 200-34). For response to exogenous PGRN, half of the wells were treated with 100 nM (final concentration) rhPGRN (AdipoGen AG-40A-0188Y) added directly to culture media. 5-h later, cells were lysed and RNA was collected using Zymo microprep kit (Zymo R1051). Genomic DNA digestion was performed on column using DNaseI (Zymo Research, E1010). cDNA was synthesized using iScript™ Reverse Transcription Supermix (Biorad 1708891) and 500 ng of RNA. qPCR was performed using SYBR Green PCR Master Mix (Applied Biosystems, 4309155). Data were acquired using 7900HT real-time system equipped with a 384-well thermal block (Applied Biosystems, Forster City, United States of America). Raw Ct values were obtained using the Sequence Detection Systems software (Applied Biosystems, version 2.4). Relative gene expression was calculated based on the delta–delta Ct method using qbase+ software (Biogazelle, version 3.0) using GAPDH as the reference gene. PCR primers: *MERTK*_F: CAGGAAGATGGGACCTCTCTGA, *MERTK*_R GGCTGAAGTCTTTCATGCACGC, *AXL*_F: GTTTGGAGCTGTGATGGAAGGC. *AXL*_R: CGCTTCACTCAGGAAATCCTCC, *GAPDH*_F: GGCCATCCACAGTCTTCTG, GAPDH_R: TCATCAGCAATGCCTCCTG. PCR was run with 3 technical replicates of each biologic replicate (independent wells of differentiated microglia).

### SPR analysis

SPR analysis was performed on a Biacore T200 Instrument (Cytiva). All analysis was performed using CM5 sensor chips activated using the standard amine capture protocol (Cytiva). To quantify binding to Mer or Axl, 10 μg/mL Protein A (21181, Pierce ThermoFisher Scientific) was first captured on the sensor chip to 2300 Resonance Units (RU). Next, 1 μg/mL of Fc-Mer (891-MR-100, R&D Systems) or Fc-Axl (154-AL-100, R&D Systems) was injected for 1 min at 20 μL/min in HBS-EP+ (BR-1006-69, Cytiva) supplemented with 1.5 mM CaCl2 and 0.1% BSA to prevent nonspecific binding. 150 RU of Fc-Mer or 140 RU of Fc-Axl was immobilized. Then, Gas6 (885-GSB-050, R&D Systems) or PGRN (AG-40A-0188Y-C050, AdipoGen) at the indicated concentration was injected at 20 μL/min for 500 s and allowed to dissociate for 1000 s. Between analyte injections, the sensor chip was completely stripped of ligand by a regeneration injection (100 mM Sodium Acetate pH 2.5, 150 mM NaCl) for 30 s at 20 μL/min. Curve fits are displayed as black lines and were determined in GraphPad Prism 9.5.1 with Nonlinear Regression using “Association then Dissociation Model” with shared values for K_on_ and K_off_.

For binding between PGRN and Gas6, Gas6 was immobilized using either an anti-Gas6 antibody (AF885-SP, R&D Systems) or anti-H6 antibody (A00186, Genscript). 10 μg/mL anti-Gas6 antibody was captured to 3000 RU. On this surface, 1 μg/mL Gas6 injected at 20 μL/min for 1 min gave a final immobilization of 135 RU. Alternatively, 10 μg/mL anti-H6 antibody of was captured to 9300 RU. On this surface, 1 μg/mL of Gas6 injected at 20 μL/min for 1 min gave a final immobilization of 205 RU. Then, the indicated concentrations of PGRN were injected at the same flow rate. Between analyte injections, the sensor chip was completely stripped of ligand and analyte by a regeneration injection (10 mM Glycine-HCL pH 2.0) for for 30 s at 20 μL/min.

For PGRN competition with Gas6 for Axl binding, 1.23 nM of Gas6 was injected alongside a titration of PGRN. Before each injection, 107 RU of Fc-Axl was immobilized onto the Protein A sensor surface. The buffer, flow rate, flow time, and regeneration conditions of the experiment were identical to those used to quantify the binary interaction between Axl and Gas6.

### Mice

Animals were maintained in full-barrier facilities free of specific pathogens on a 12-hour light/dark cycle with food and water *ad libitum*. Experiments were conducted in compliance with the Institutional Animal Care and Use Committee at the University of California, San Francisco (AN173162-02). We have complied with all relevant ethical regulations for animal use. We backcrossed *Mertk* KO^2^ (JAX 011122) and *Axl* KO^2^ (JAX 011121) to C57Bl/6 WT and *Grn* KO mice^22^ (JAX 013175) to ensure all mice were on the C57Bl/6 background. Genotype was confirmed by gene-specific PCR primers and Western blot. Mice were deeply anesthetized with Avertin before cardiac perfusion with 0.9% saline. Hemibrains were removed and either snap-frozen on dry ice for single-cell studies or postfixed for 48 h in 4% paraformaldehyde and cryoprotected in 30% sucrose for cryosectioning.

### Immunohistochemistry

Staining and quantification of mouse brain tissue was done by NeuroScience Associates. Fixed hemibrains were treated overnight with 20% glycerol and 2% dimethylsulfoxide to prevent freeze artifacts. The specimens were then embedded in a gelatin matrix using MultiBrain® Technology (NeuroScience Associates, Knoxville, TN). The blocks were rapidly frozen, after curing by immersion in 2-Methylbutane chilled with crushed dry ice and mounted on a freezing stage of an AO 860 sliding microtome. The multi-brain blocks were sectioned coronally at 30 μm and stored in Antigen Preserve solution (50% PBS pH7.0, 50% Ethylene Glycol, 1% Polyvinyl Pyrrolidone). For Iba1 staining, 4 free-floating sections throughout the rostral-caudal axis of the thalamus were rinsed with Tris buffered saline (TBS) 3 times, permeabilized with TBS with 0.018% Triton X-100 for 30 min, then 3% hydrogen peroxide for 30 min at room temperature. Tissues were incubated with rabbit Iba1 (Abcam ab178846) at 1:75,000 overnight at room temperature. After 3 rinses in TBS sections were incubated with biotinylated secondary antibody (goat anti rabbit, VectorLabs BA-1000) 1:1000 for 1 h at room temperature. After 3 additional rinses in TBS, we applied Vector Lab’s ABC solution (avidin-biotin-HRP complex; details in instruction for VECTASTAIN® Elite ABC, Vector, Burlingame, CA). The sections were again rinsed 3 times in TBS, then treated with diaminobenzidine tetrahydrochloride (DAB), nickel (II) sulfate hexahydrate and hydrogen peroxide to create a visible reaction product. Following 3 further TBS rinses, the sections were mounted on gelatin coated glass slides and air dried. The slides were dehydrated in alcohol of increasing percentages, cleared in xylene and cover slipped. Sections were imaged on a TissueScope LE120 whole slide scanning system (Huron Digital Pathology). Analysis was performed by circling the thalamus through the rostral-caudal extent and Iba1+ count was quantified using an algorithm developed by Aiforia after training on separate brain sections. Iba+ count was normalized to the section area (mm^2^) and averaged per mouse.

### Reporting summary

Further information on research design is available in the Nature Portfolio Reporting Summary linked to this article.

## Supplementary information

Supplementary Information

Description of additional supplementary files

Supplementary Data 1-5

Reporting Summary

## Data Availability

All RNA-seq data are deposited to the Gene Expression Omnibus (GEO) under accession number GSE250280. Raw human CSF proteomic data are available upon reasonable request from qualified investigators (https://www.allftd.org/data). Certain data elements may be restricted due to the potential for identifiability in the context of the sensitive nature of genetic data.

## Code availability

All custom code used for snRNAsea data analysis have been archived at Zenodo (10.5281/zenodo.14941325) and are directly available at https://github.com/lifan36/Clelland-Fan-FTD-2023.
