# Supplementary material for: Opposing role of phagocytic receptors MERTK and AXL in Progranulin deficient FTD
Source: Commun Biol. 2025 Jul 1;8:971. doi: 10.1038/s42003-025-08368-2 (PMC12218935; doi:10.1038/s42003-025-08368-2)
Supplement: Supplementary file 1 — Supplementary Information [file 42003_2025_8368_MOESM1_ESM.pdf]

Supplemental Figures & Methods

Opposing role of phagocytic receptors MERTK and AXL in Progranulin deficient FTD

Claire Dudley Clelland[1,2]*#, Li Fan[3]*, Rowan Saloner[2], Jon Iker Etchegaray[4], Chad Richard Altobelli[5], Sally Salomonsson[1], Alisha M. Maltos[4], Aradhana Sachdev[4], Jingjie Zhu[3], Se-In Lee[3], Yaqiao Li[4], Yungui Zhou[4], David Le[4], Chao Wang[4], Gillian Carling[3], Lay Kodama[4], Faten Sayed[4], Jaun A. Perez-Bermejo[4], Ethan G. Geier[1,2], Jennifer S. Yokoyama[1,2], Howie Rosen[2], Alissa L. Nana[2], Salvatore Spina[2], Lea T. Grinberg[2], William W. Seeley[2], Fanny Elahi[2], Adam L. Boxer[2], Michelle R. Arkin[5], Li Gan[3]#.

[1]Weill Institute for Neurosciences, University of California San Francisco, San Francisco, California, USA
[2]Memory & Aging Center, Department of Neurology, University of California San Francisco, San Francisco, California, USA
[3]Helen and Robert Appel Alzheimer's Disease Research Institute, Brain and Mind Research Institute, Weill Cornell Medicine, New York, NY USA
[4]Gladstone Institutes, San Francisco, California, USA
[5]Department of Pharmaceutical Chemistry and Small Molecule Discovery Center; University of California, San Francisco, California, USA

* These authors contributed equally
# Correspondence sent to: claire.clelland@ucsf.edu; lig2033@med.cornell.edu

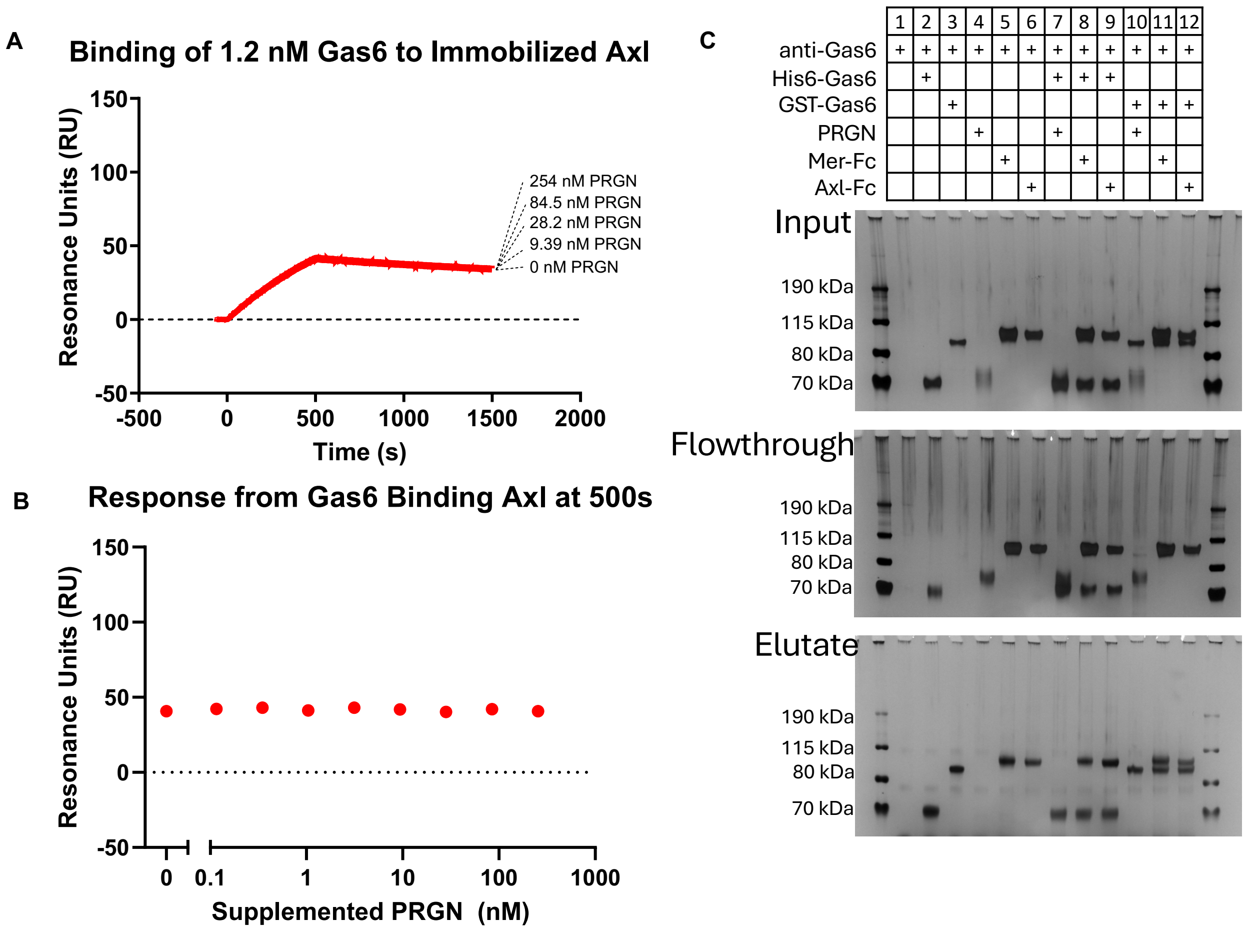

Supplemental Fig. 1. PGRN does not bind GAS6 in SPR and immunoprecipitation assays. (A,B) Surface plasmon resonance (SPR) showing a constant 1.2 nM injection of GAS6 binds to immobilized AXL with similar kinetics and maximum response regardless of supplemented PGRN. This indicates that PGRN does not compete or cooperate with AXL for GAS6 binding. The theoretical R_{max} for this interaction is 135. **(C)** Beads loaded with an anti-GAS6 antibody bind both His6-GAS6 (lane 2) and GST-GAS6 (lane 3) but not PGRN alone (lane 4). When PGRN is introduced to GAS6 immobilized beads (lane 7 & 10), it flows through without binding GAS6 and is not present in the eluate. First and last lanes = ladder. Gels were visualized using a Pierce™ Silver Stain Kit.

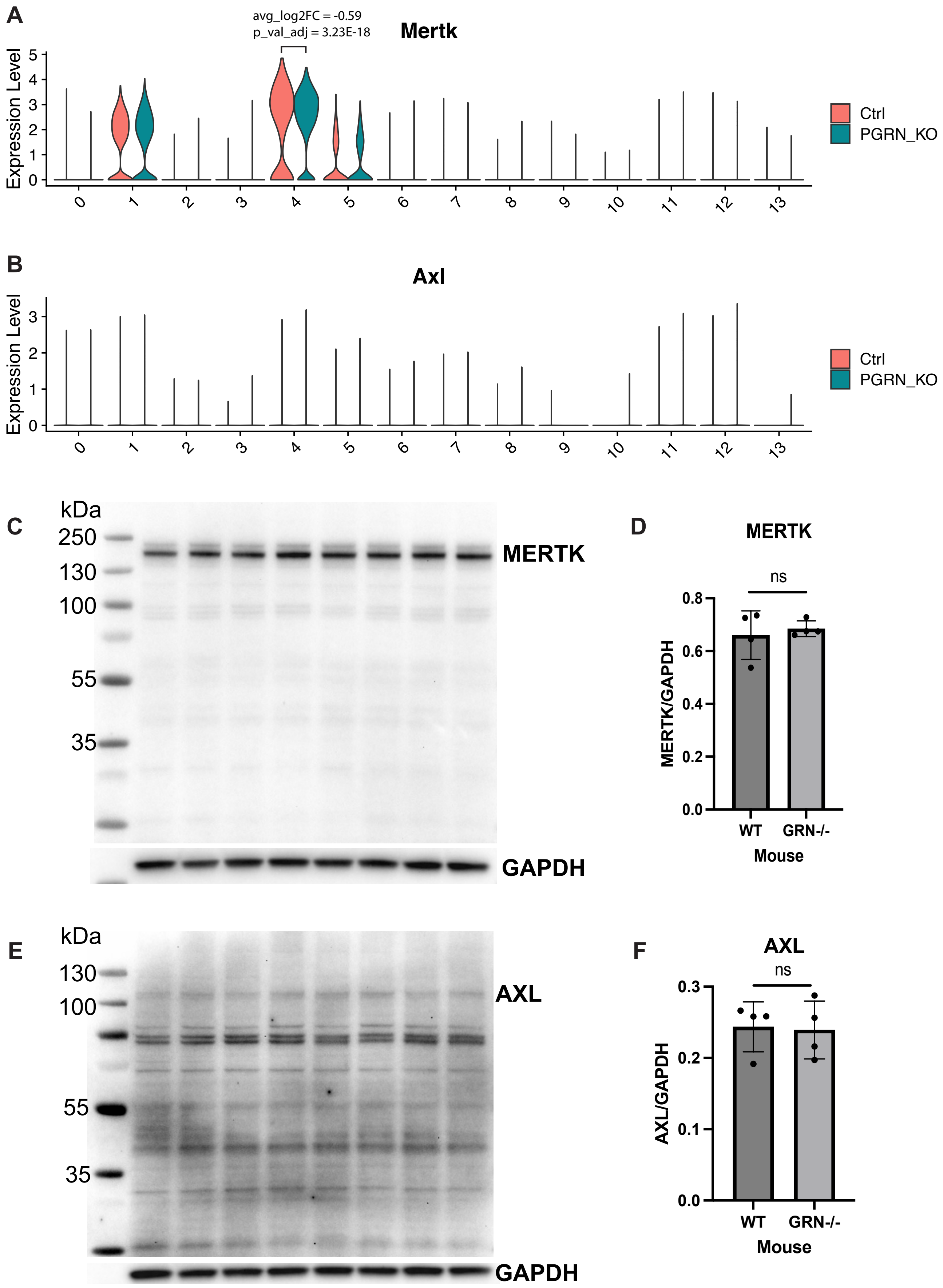
A
avg_log2FC = -0.59
p_val_adj = 3.23E-18
Mertk
Expression Level
Ctrl
PGRN_KO
B
Axl
Expression Level
Ctrl
PGRN_KO
C
kDa
250
130
100
55
35
MERTK
GAPDH
D
MERTK
ns
MERTK/GAPDH
WT
GRN-/-
Mouse
E
kDa
130
100
55
35
AXL
GAPDH
F
AXL
ns
AXL/GAPDH
WT
GRN-/-
Mouse

Supplemental Fig. 2. *Mertk* and *Axl* RNA and protein expression in *Grn* KO mice compared to WT controls. (A,B) Violin plot showing expression of *Mertk* (A) and *Axl* (B) mRNA in post-mortem mouse thalamus. Cell type numbers correspond to Fig. 4A. **(C-F)** Mertk and Axl protein levels in mouse thalamus. MERTK (C,D) was equivalent in GRN KO mice compared to WT (unpaired 2-tailed T-test p=0.63). AXL (E,F) levels were also equivalent between GRN KO and WT (unpaired 2-tailed T-test p=0.88). Error bars = SEM.

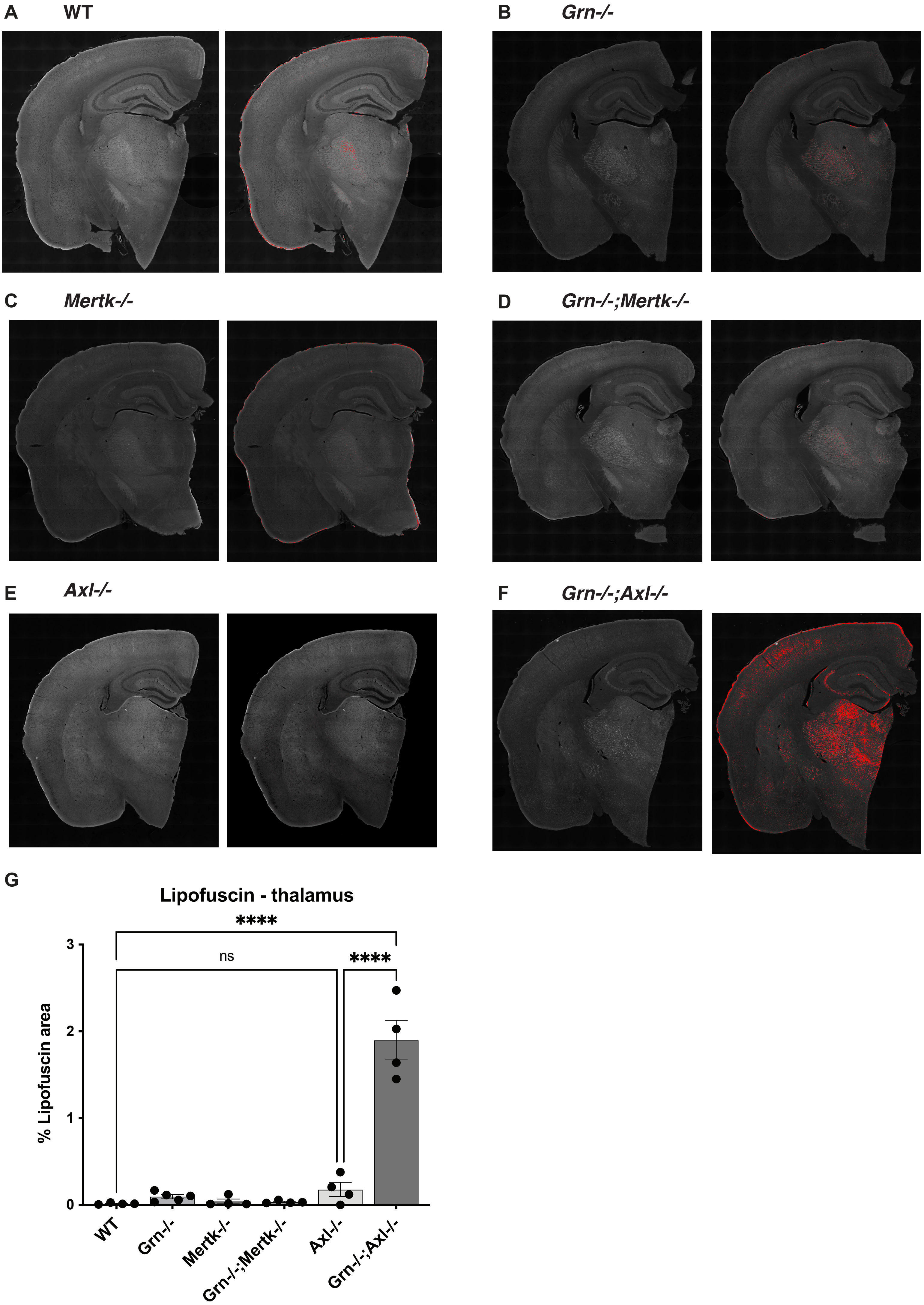
A
WT
B
Grn-/-
C
Mertk-/-
D
Grn-/-;Mertk-/-
E
Axl-/-
F
Grn-/-;Axl-/-
G
Lipofuscin - thalamus
****
ns
****
% Lipofuscin area
0
1
2
3
WT
Grn-/-
Mertk-/-
Grn-/-;Mertk-/-
Axl-/-
Grn-/-;Axl-/-

Supplemental Fig. 3. Loss of *Axl* worsens lipofuscin accumulation in *Grn* knock-out mice, whereas loss of *Mer* does not affect lipofuscin accumulation. (A-F) Auto fluorescent signal (*i.e.,* lipofuscin) in unstained mouse brains is shown in grey scale (left image) and pseudo-colored in red (right image). Lipofuscin was greatest in *Grn*-/-;*Axl*-/- (one-way ANOVA $F(5,19)=60.2$, $p<0.0001$, Tukey's multiple comparison test ****$p<0.0001$). Mild lipofuscinosis was observed in *Grn*-/- and *Grn*-/-;*Mertk*-/- and none or nearly none in WT, *Mertk*-/- and *Axl*-/-. Error bars = SEM.

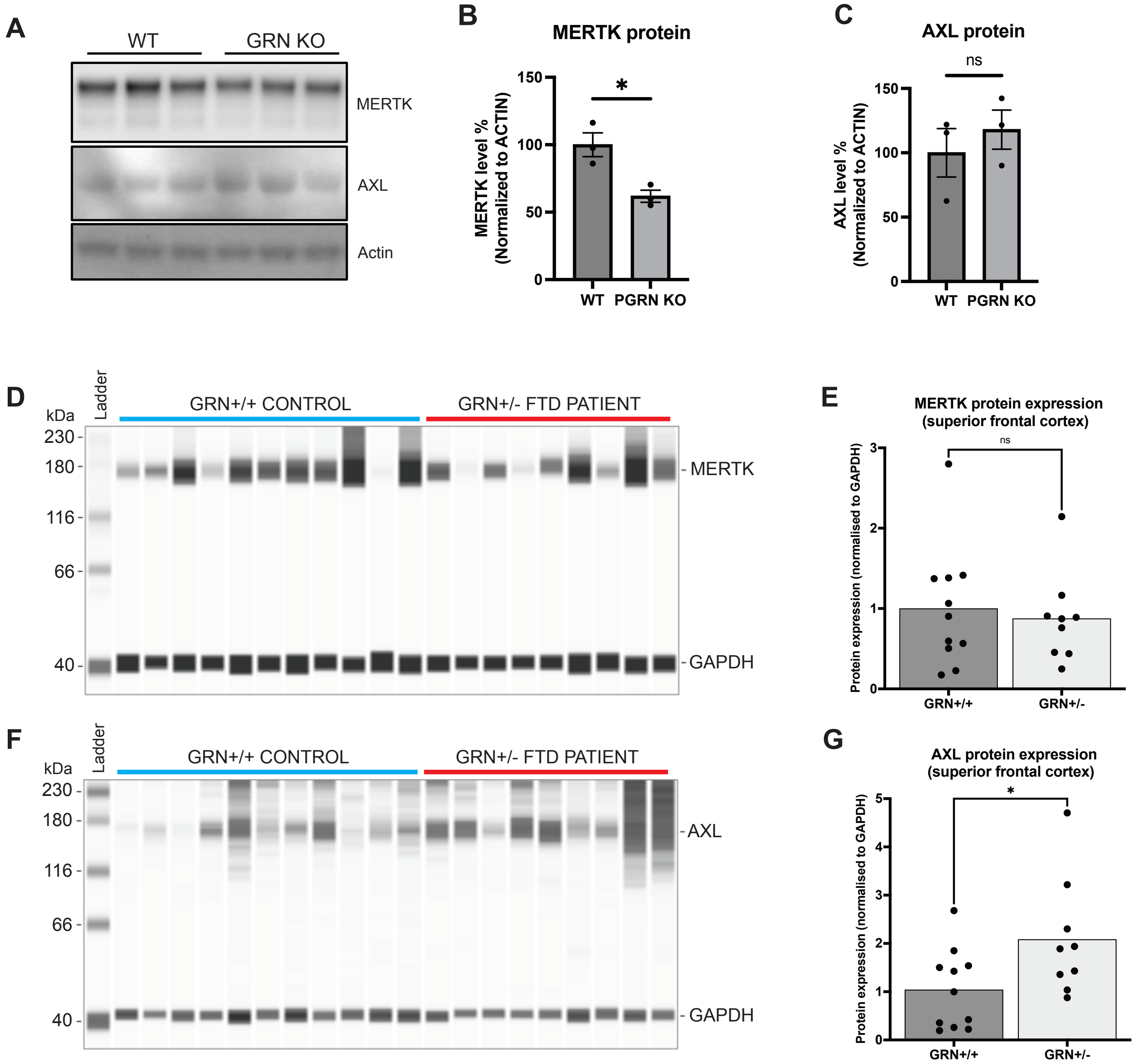

Supplemental Fig. 4. Divergence of MERTK and AXL protein levels between human induced microglia and post-mortem brain tissue. (A-C) MERTK and AXL protein levels in induced microglia *in vitro* determined by Western blot. MERTK **(A,B)** was significantly decreased in 28-day old GRN KO induced microglia compared to WT (2-tailed T-test p=0.01) while AXL **(A,C)** levels were not significantly different between GRN KO and WT (2-tailed T-test p=0.50). **(D,E)** Simple Western of MERTK levels from post-mortem superior frontal cortex did not differ between *GRN+/-* FTD patient and controls (Mann Whitney U p=0.71). **(F,G)** Simple Western of AXL levels were significantly increased in *GRN+/-* FTD patients (unpaired, two-tailed t-test, p<0.05). Error bars = SEM. *p < 0.05.

Supplemental Methods

GAS6 and PGRN competition assay by SPR. See main text methods for full description of SPR. For PGRN competition with Gas6 for Axl binding, 1.23 nM of Gas6 was injected alongside a titration of PGRN. Before each injection, 107 RU of Fc-Axl was immobilized onto the Protein A sensor surface. The buffer, flow rate, flow time, and regeneration conditions of the experiment were identical to those used to quantify the binary interaction between Axl and Gas6.

Immunoprecipitation. To begin, a large batch of ProteinG Dynabeads (10003D, Invitrogen) were loaded with anti-hGas6 antibody (AF885-SP, R&D Systems). 55 uL of Dynabeads were added to a microfuge tube, placed on a magnetic rack, and supernatant was removed. The beads were washed once in 200 uL immunoprecipitation buffer composed of HBS-EP supplemented with 1.5mM $CaCl_2$ and 1mM DTT. The beads were then resuspended in 200uL of buffer supplemented with 14.5 ug anti-hGas6 antibody and allowed to incubate for 10min @ 23C. The beads were washed twice with 200uL buffer, then resuspended and split between 11 tubes. To each tube was added 40uL of the appropriate protein extract mixture composed of His6-Gas6 (885-GSB-050, R&D Systems), GST-Gas6 (H00002621-P01, Abnova), PGRN (AG-40A-0188Y-C050, AdipoGen), Fc-Mer (891-MR-100, R&D Systems) and/or Fc-Axl (154-AL-100 , R&D Systems). 10uL of these protein mixtures was saved for the input gel. The tubes were then inverted on a rotating shaker for 10 min at 23C to allow binding between Gas6 and the beads. The beads were then collected and the supernatant saved for the flowthrough gel. The beads were washed with 150uL of buffer three times. To elute bound protein, the beads were incubated with 1x NuPAGE sample buffer supplemented with 10% BME at 90C for 10min. The protein samples were run on NuPAGE™ Bis-Tris Mini Protein Gels, 4–12% at 180v for 80min, then visualized with a Pierce Silver Stain Kit according to the manufacturer protocol.

Mertk and Axl protein quantification from mouse brain. *Protein lysate preparation*: 30 mg thalamus was homogenized in 300ul RIPA buffer (Thermo, Cat # 89901) with complete Protease Inhibitors (Roche, Cat # 11697498001) and Halt Phosphatase Inhibitors (Thermo, Cat # 1862495) by handheld homogenizer. Protein lysates were collected after centrifugation at 20000 x g for 15 mins at 4°C. Protein concentrations were measured by using Pierce BCA Protein Assay Kit (Thermo, Cat # 23225). *Western blot:* 30 ug thalamus lysates mixed with 4x LDS sample buffer (Invitrogen, Cat# NP0008) containing 20% beta-mercaptoethanol were boiled at 95 ^{o}C for 5 minutes and run on 10-well 4-12% Bis-Tris gels (Invitrogen) using MES buffer (Invitrogen, Cat# NP0002) for 1.5 hour. Proteins were transferred from gel onto PVDF membrane at 0.36A for 2 hours. Membranes were blocked in 5% skim milk for 1 hour at room temperature and incubated with anti-Mer (1:500, R&D,Cat# AF591) and anti-Axl (1:500 R&D, Cat# AF854) antibodies in 1% skim milk overnight in cold room and followed by washing 3 times for 5 min each in TBS with 0.01% triton X-100 (TBST). Membranes were incubated with appropriate HRP-conjugated secondary antibody in 1% skim milk for 1 hour at room temperature, washed in TBST, developed by ECL substrate (Biorad, Cat# 1705060) and imaged using Bio-Rad imager. Membranes were also incubated with HRP-conjugated GAPDH (GeneTex, Cat# GTX627408-01) for 1 hour at room temperature before washing in TBST, ECL development and imaging. The images were quantified by using Bio-Rad Image Lab software.

Lipofuscin imaging and quantification. Post-fixation treatment and sectioning of mouse brains were detailed in the main text methods under "Immunohistochemistry." 30 µm sections were rinsed with Tris buffered saline (TBS) then treated with 1:15,000 Hoescht (Thermo H3575) in TBS for 15 min followed by 3 additional rinses in TBS, mounted on gelatin coated glass slides, then air dried. The stained slides were dehydrated and cleared per the main text protocol. Hoechst-stained mouse brain sections were scanned using an Olympus VS200 Research Slide Scanner (Olympus) at 20x resolution (0.274um/pixel resolution). The FITC (488) channel was scanned for evaluation of lipofuscin. Analysis was performed by circling the thalamus through the rostral-caudal extent and %area autofluorescence was quantified.

iPSC induced microglia differentiation, Mertk and Axl quantification corresponding to Supplemental Fig. 4. *Human iPSC Culture:* Human iPSCs and isogenic GRN KO iPSCs were obtained from Jackson Laboratory (JAX JIPSC001644).. These iPSCs were cultured in hESC-Qualified Matrigel (Corning)-coated 6-well plates using mTeSR plus media (STEMCELL). At the cell confluence 80% to 90%, iPSCs were detached using ReLeSR (STEMCELL). The dissociated cells were seeded to a new Matrigel-coated 6-well plate with mTeSR plus media containing 10 μM ROCK inhibitor (Tocris).

Differentiation of Human iPSC-derived Hematopoietic Progenitor Cells (HPCs)
To generate the hiPSC-deirved HPCs, we used the STEMdiff Hematopoietic kit (STEMCELL). Briefly, 180 iPSC colonies were seeded Matrigel-coated 6-well plate with mTeSR plus containing 10 μM ROCK inhibitor. At Day 0, the media was replaced with Basal media containing Sup A. At Day 3, the media was changed with Basal media containing Sup B. Half of the media was replaced every other day. On Day 13, HPCs were detached with pipetting and then collected the cell by centrifugation at 300x g for 5 minutes.

Differentiation of HPCs to Human iPSC-derived Microglia like-cells (iMGLs)
HPCs were seeded to a Matrigel-coated 6-well plate at a density of 700,000 cells per well. For the differentiation, we used Microglia Basal media including DMEM/F12 (Gibco), B27 (Gibco), N2 (Gibco), GlutaMAX (Gibco), NEAA (Sigma Aldrich), Monothioglycerol (Sigma Aldrich), insulin-transferrin-selenite (Gibco), and insulin (Sigma Aldrich). At Day 0, HPCs were cultured with Microglia Basal media containing 100ng/ml of IL-34 (Peprotech), 50ng/ml of TGF-β1 (STEMCELL), and 25 ng/ml of M-CSF (R&D Systems). 1ml of the basal media with 3 cytokines was added every other day. On Day 12, 7ml of the media was discarded from the plate, and 1ml of the media was added every other day until Day 24. At Day 25, 7ml of media was discarded and added 1ml of the media additionally containing 2 more cytokines: 100 ng/ml of CD200 (Novo protein) and 100ng/ml of Cx3CL1 (STEMCELL). On Day 28, iMGLs were transferred to new plates for experiments.

Human MERTK and AXL protein quantification from post-mortem tissue by Simple Western. We performed protein quantification by streptavidin-based Simple Western capillary reaction (WES; Bio-Techne) according to the manufacturers' protocol (Jess & Wes Separation Module SM1001 to SM1012), with the following specifications: Protein was collected from post-mortem human brain tissue in RIPA buffer with protease inhibitor, sonicated for 5 min, and denatured at 90°C for 10 min. Each sample was mixed with Fluorescent 5X Master Mix and diluted in 0.1X Sample Buffer (EZ Standard Pack PS-ST01EZ-8) to a final protein concentration of 0.22 µg/µl, followed by denaturing at 95°C for 5 min. 3 µl of this mix was loaded per sample onto a 12-230 kDa separation module (ProteinSimple SM-W004). Primary antibodies were rabbit anti-MERTK (Abcam, ab52968) at a 1:1000 dilution, goat anti-AXL (R&D Systems, AF154) at a 1:100 dilution, and rabbit anti-GAPDH (Abcam, ab9485) at 1:250 dilution (total volume 10 µl per lane). Duplexed secondaries included 9.5 µl of anti-goat (ProteinSimple, DM-006) and 0.5 µl of 20X anti-rabbit HRP conjugate (ProteinSimple, 043-426) per lane. Reaction times: 25 min separation time at 375 V, 5 min antibody dilutant time, 30 min primary antibody, 30 min secondary antibody; quantification at 4 seconds of detection (high dynamic range). Under these optimized conditions, each antibody produced a peak at 176 kDa (MERTK), 165 kDa (AXL) or 42 kDa (GAPDH). Area under the curve was quantified for each peak and target AUC was normalized to reference GAPDH AUC for each sample. Two technical replicates for each patient were averaged and compared to the average protein expression of neurologically unaffected controls.
