# Supplementary material for: Opposing role of phagocytic receptors MERTK and AXL in Progranulin deficient FTD
Source: Commun Biol. 2025 Jul 1;8:971. doi: 10.1038/s42003-025-08368-2 (PMC12218935; doi:10.1038/s42003-025-08368-2)
Supplement: Supplementary file 2 — Description of additional supplementary files [file 42003_2025_8368_MOESM2_ESM.docx]

Description of Additional Supplementary Files 1

**File name**: Supplementary Data 1: Patient clinical information

**Description**: Table of characteristics pertaining to the 24 donors whose brain tissues were used in this manuscript. Samples used for single nuclei RNAseq and western blot indicated.

**File name**: Supplementary Data 2: DEGs of GRN+/- vs Ctrl in each cell type in patient brains

**Description**: Table summarizing differential expression analysis between human GRN+/- cells vs Ctrl cells for each brain cell type.

**File name**: Supplementary Data 3: Markers for the microglia subpopulations isolated from human PGRN+/- and control tissues

**Description**: Table summarizing microglial subpopulation marker genes.

**File name**: Supplementary Data 4: Cluster marker genes in mouse snRNAseq

**Description**: Cluster marker genes which are up-regulated by at least 1-fold against other cluster cells with p_val_adj ≤ 0.05 from snRNAseq of 10-12-month-old WT control, single KO mice (Grn-/-, Mertk-/- and Axl-/- mice) and double KO mice (Grn-/-;Mertk-/-, Grn-/-;Axl-/-).

**File name**: Supplementary Data 5: DEGs of mouse microglia cluster 2 vs cluster 1

**Description**: Table of differential expression analysis between microglia cluster 2 vs cluster 1 from snRNAseq of 10-12-month-old WT control, single KO mice (Grn-/-, Mertk-/- and Axl-/- mice) and double KO mice (Grn-/-;Mertk-/-, Grn-/-;Axl-/-).
