# Supplementary material for: Opposing role of phagocytic receptors MERTK and AXL in Progranulin deficient FTD
Source: Commun Biol. 2025 Jul 1;8:971. doi: 10.1038/s42003-025-08368-2 (PMC12218935; doi:10.1038/s42003-025-08368-2)
Supplement: Supplementary file 4 — Reporting Summary [file 42003_2025_8368_MOESM4_ESM.pdf]

nature portfolio

Corresponding author(s): *Double-anonymous peer review submissions: write DAPR and your manuscript number here instead of author names.*

Last updated by author(s): May 20, 2025

Reporting Summary

Nature Portfolio wishes to improve the reproducibility of the work that we publish. This form provides structure for consistency and transparency in reporting. For further information on Nature Portfolio policies, see our Editorial Policies and the Editorial Policy Checklist.

Statistics

For all statistical analyses, confirm that the following items are present in the figure legend, table legend, main text, or Methods section.

n/a	Confirmed	
☐	☒	The exact sample size (*n*) for each experimental group/condition, given as a discrete number and unit of measurement
☐	☒	A statement on whether measurements were taken from distinct samples or whether the same sample was measured repeatedly
☐	☒	The statistical test(s) used AND whether they are one- or two-sided<br>*Only common tests should be described solely by name; describe more complex techniques in the Methods section.*
☐	☒	A description of all covariates tested
☐	☒	A description of any assumptions or corrections, such as tests of normality and adjustment for multiple comparisons
☐	☒	A full description of the statistical parameters including central tendency (e.g. means) or other basic estimates (e.g. regression coefficient) AND variation (e.g. standard deviation) or associated estimates of uncertainty (e.g. confidence intervals)
☐	☒	For null hypothesis testing, the test statistic (e.g. *F*, *t*, *r*) with confidence intervals, effect sizes, degrees of freedom and *P* value noted<br>*Give P values as exact values whenever suitable.*
☒	☐	For Bayesian analysis, information on the choice of priors and Markov chain Monte Carlo settings
☒	☐	For hierarchical and complex designs, identification of the appropriate level for tests and full reporting of outcomes
☐	☒	Estimates of effect sizes (e.g. Cohen's *d*, Pearson's *r*), indicating how they were calculated

Our web collection on statistics for biologists contains articles on many of the points above.

Software and code

Policy information about availability of computer code

Data collection	No software was used to collect data.
Data analysis	All custom codes used for snRNAsea data analysis are available at https://github.com/lifan36/Clelland-Fan-FTD-2023.<br>For single-nuclei RNA sequencing, gene counts were acquired by aligning reads to the human genome (hg38) mouse genome (mm10) using Cell Ranger software (version 3.1.0) by 10x Genomics. Potential doublet cells were predicted and eliminated for each sample using DoubletFinder. Normalization and clustering were carried out with Seurat package version 3.0.182. Gene network and functional enrichment analysis were executed using QIAGEN's Ingenuity® Pathway Analysis (IPA®) available at www.qiagen.com/ingenuity.<br>Statistical analyses were conducted using GraphPad Prism 8 from GraphPad in San Diego, California, and R from the R Foundation for Statistical Computing in Vienna, Austria. Data visualization was performed using GraphPad and the R package ggplot2.

For manuscripts utilizing custom algorithms or software that are central to the research but not yet described in published literature, software must be made available to editors and reviewers. We strongly encourage code deposition in a community repository (e.g. GitHub). See the Nature Portfolio guidelines for submitting code & software for further information.

Data

Policy information about availability of data

All manuscripts must include a data availability statement. This statement should provide the following information, where applicable:

- Accession codes, unique identifiers, or web links for publicly available datasets
- A description of any restrictions on data availability
- For clinical datasets or third party data, please ensure that the statement adheres to our policy

All data associated with this study are in the paper or in the Supplementary Materials. All RNA-seq data are deposited to the Gene Expression Omnibus (GEO) under accession number GSE250280. Raw human CSF proteomic data are available upon reasonable request from qualified investigators (https://www.allftd.org/data). Certain data elements may be restricted due to the potential for identifiability in the context of the sensitive nature of genetic data.

Research involving human participants, their data, or biological material

Policy information about studies with human participants or human data. See also policy information about sex, gender (identity/presentation), and sexual orientation and race, ethnicity and racism.

Reporting on sex and gender	Sex is reported in Supplemental Data and in previously published cohort.
Reporting on race, ethnicity, or other socially relevant groupings	n/a
Population characteristics	population characteristics are present in Supplemental Data 1.
Recruitment	The inclusion of patients is described in methods.
Ethics oversight	IRB review board information included in methods.

Note that full information on the approval of the study protocol must also be provided in the manuscript.

Field-specific reporting

Please select the one below that is the best fit for your research. If you are not sure, read the appropriate sections before making your selection.

☒ Life sciences ☐ Behavioural & social sciences ☐ Ecological, evolutionary & environmental sciences

For a reference copy of the document with all sections, see nature.com/documents/nr-reporting-summary-flat.pdf

Life sciences study design

All studies must disclose on these points even when the disclosure is negative.

Sample size	FTD from GRN gene mutation carriers is a rare disease and brain tissue from these patients hard to obtain. Our sample size was determined by the tissue available to us that passed QC metrics for each assay (as indicated in the methods) and by the available CSF proteomics from ALLFTD.
Data exclusions	The only data excluded was tissue that failed to pass RNA or protein quality metrics or if there was missing pre-death clinical values. This was noted in the methods and in Supplemental Data 1.
Replication	Where possible we replicate data (such as the iPSC-induced neuron experiments) and all replicates are shown.
Randomization	N/A to this study.
Blinding	Scientists who performed single-cell collection, protein quantification, and histology were all blinded to the experimental condition. Only those analyzing the data had the group assignments.

Reporting for specific materials, systems and methods

We require information from authors about some types of materials, experimental systems and methods used in many studies. Here, indicate whether each material, system or method listed is relevant to your study. If you are not sure if a list item applies to your research, read the appropriate section before selecting a response.

Materials & experimental systems

n/a	Involved in the study	
☐	☒	Antibodies
☐	☒	Eukaryotic cell lines
☒	☐	Palaeontology and archaeology
☐	☒	Animals and other organisms
☐	☒	Clinical data
☒	☐	Dual use research of concern
☒	☐	Plants

Methods

n/a	Involved in the study	
☒	☐	ChIP-seq
☒	☐	Flow cytometry
☒	☐	MRI-based neuroimaging

Antibodies

Antibodies used	Primary antibodies were rabbit anti-MERTK (Abcam, ab52968) at a 1:1000 dilution, goat anti-AXL (R&D Systems, AF154) at a 1:100 dilution, and rabbit anti-GAPDH (Abcam, ab9485) at 1:250 dilution (total volume 10 µl per lane). Duplexed secondaries included 9.5 µl of anti-goat (ProteinSimple, DM-006) and 0.5 µl of 20X anti-rabbit HRP conjugate (ProteinSimple, 043-426) per lane.
Validation	We used KO-validated MERTK and AXL antibodies, as indicated by the manufacturer website.

Eukaryotic cell lines

Policy information about cell lines and Sex and Gender in Research

Cell line source(s)	PGRN KO iPSCs(25) were generated and validated by the Conklin lab at Gladstone Institutes in a non-diseased cell line (WTC). We cite the previous validation for this line.
Authentication	See above. In addition, we karyotype all lines before experimentation. We confirmed the line was a KO by WB. We test for mycoplasma monthly.
Mycoplasma contamination	We test monthly for mycoplasma for every cell line. All tests were negative.
Commonly misidentified lines (See ICLAC register)	N/A

Animals and other research organisms

Policy information about studies involving animals; ARRIVE guidelines recommended for reporting animal research, and Sex and Gender in Research

Laboratory animals	We backcrossed Mertk KO2 (JAX 011122) and Axl KO2 (JAX 011121) to C57Bl/6 WT and Grn KO mice(22) (JAX 013175) to ensure all mice were on the C57Bl/6 background. Genotype was confirmed by gene-specific PCR primers and Western blot.
Wild animals	WT literrmates were used.
Reporting on sex	Sex is reported in the methods.
Field-collected samples	N/A
Ethics oversight	Statement from methods: "Experiments were conducted in compliance with the Institutional Animal Care and Use Committee at the University of California, San Francisco (AN173162-02)."

Note that full information on the approval of the study protocol must also be provided in the manuscript.

Clinical data

Policy information about clinical studies
All manuscripts should comply with the ICMJE guidelines for publication of clinical research and a completed CONSORT checklist must be included with all submissions.

Clinical trial registration	N/A
Study protocol	Not a clinical trial.
Data collection	The study protocol was approved by the UCSF Committee on Human Research Institutional Review Board.
Outcomes	Clinical outcome rating was included in Supplemental Data 1.

Plants

Seed stocks

N/A

Novel plant genotypes

Describe the methods by which all novel plant genotypes were produced. This includes those generated by transgenic approaches, gene editing, chemical/radiation-based mutagenesis and hybridization. For transgenic lines, describe the transformation method, the number of independent lines analyzed and the generation upon which experiments were performed. For gene-edited lines, describe the editor used, the endogenous sequence targeted for editing, the targeting guide RNA sequence (if applicable) and how the editor was applied.

Authentication

Describe any authentication procedures for each seed stock used or novel genotype generated. Describe any experiments used to assess the effect of a mutation and, where applicable, how potential secondary effects (e.g. second site T-DNA insertions, mosiacism, off-target gene editing) were examined.
